# Evaluation of the growth, enzymatic activity, electrolyte leakage, and phytoremediation efficiency of *Conocarpus erectus* under cadmium and lead stress

**DOI:** 10.3389/fpls.2024.1466697

**Published:** 2024-09-30

**Authors:** El-Sayed Mohamed El-Mahrouk, Shereen Mostafa Eldawansy, Ahmed Mohamed El-Tarawy, Hayam Mohamed Aly Ebrahim, Eman Abdelhakim Eisa, Andrea Tilly-Mándy, Péter Honfi

**Affiliations:** ^1^ Horticulture Department, Faculty of Agriculture, Kafrelshaikh University, Kafrelsheik, Egypt; ^2^ Floriculture and Landscape Gardens Department, Horticulture Research Institute Alex. Branch (Antoniadis), Alexandria, Egypt; ^3^ Botanical Gardens Research Department, Horticulture Research Institute, Agricultural Research Center (ARC), Giza, Egypt; ^4^ Floriculture and Dendrology Department, Hungarian University of Agriculture and Life Science (MATE), Budapest, Hungary

**Keywords:** phytoremediation, cadmium, lead, pollution, *Conocarpus erectus*

## Abstract

Contamination of agricultural soil by heavy metals poses a significant threat to soil quality and crop yields. Using plants as a natural remediation approach attracts researchers’ attention around the world. A 16-month pot experiment was conducted using *Conocarpus erectus* in a randomized complete block design. The growth, enzymatic activity, electrolyte leakage, and remediation potential were estimated under Cd nitrate]40 low (L), 60 medium (M), 80 high (H) mg/kg soil [and Pb nitrate]400 (L), 700 (M), 1,000 (H) mg/kg soil [applied individually and in combination. *Conocarpus erectus* demonstrated a good tolerance (over 70%) against lower and medium cadmium (Cd) and lead (Pb) levels and a medium resistance against high Cd and Pb levels, with a survival rate of 100% under all the treatments used. The most negative treatment on the growth traits and tolerance of *C. erectus* was (H) Cd and (H) Pb, which reduced plant height; chlorophyll index; dry weights of the leaves, stems, and roots; root length; and tolerance index of biomass and roots by 25.87%, 48.97%, 50.56%, 47.25%, 58.67%, 50.18%, 51.00%, and 50% in comparison to the respective control, consecutively. Relative to the control, all Cd and Pb applications increased polyphenol oxidase (PPO), peroxidase (POD), and catalase (CAT) activities, and the increment was parallel up to medium Cd and Pb levels and then decreased with their high levels but still higher than the control. Electrolyte leakage (EL) was upheaved by raising the levels of Cd and Pb, and it reached the maximum (52.79%) at the (H) Cd (H) Pb treatment. Cd and Pb in the leaves, stems, and roots were boosted by raising their levels in the treatments. *Conocarpus erectus* is considered a phytoextractor for the Cd levels used because the bioconcentration factor of the stem (BCF_s_) and the translocation factor (TF) of Cd were >1, and it is a suitable plant for Pb phytoextraction at (L) Pb, (M) Pb, and (M) Cd (M) Pb levels because its Pb BCF_s_ and bioconcentration factor of the root (BCF_r_) were <1 and its Pb TF was >1. On the other hand, *C. erectus* is considered a phytostabilizator for Pb at (H) Pb, (L) Cd, (L) Pb, and (H) Cd (H) Pb levels because its Pb BCF_s_, BCF_r_, and TF were <1.

## 1 Introduction

Heavy metals or trace element ions, derived from anthropogenic activities and industrial development, represent some of the most severe environmental contaminants today. Soil and aquatic pollution by heavy metals pose significant threats to human health, aquatic ecosystems, and agriculture (Overesch et al., 2007). Additionally, heavy metal contamination of agricultural soil is a major global challenge. The primary visual effects of heavy metal poisoning in plants include chlorosis, reduced growth, impaired respiration and nitrogen metabolism, reduced water and nutrient absorption, and decreased photosynthetic rate (Benavides et al., 2005; Wang et al., 2008). Cadmium (Cd) and lead (Pb) are widely recognized as the most prominent heavy metals (Redovnikovic et al., 2017). The negative effects of Cd include reduced biomass production, decreased enzyme activity, disruption of soluble protein metabolism, and increased generation of reactive oxygen species (ROS), ultimately hindering plant growth (Kieffer et al., 2009; Ali et al., 2014a; EF et al., 2015). Moreover, Cd adversely affects soil microorganisms, leading to reduced organic matter decomposition and decreased soil nutrient availability, which further inhibits plant growth (Qiu et al., 2022).

Pb causes chlorosis and reduces seed germination, growth and biomass, photosynthetic rate, essential element absorption, and water transport. It also alters membrane permeability and increases ROS production in plants, interfering with enzyme activities within plant cells (Ali et al., 2014b; Saxena et al., 2020). According to Wyszkowska et al. (2024), Pb at concentrations of 800 mg/kg significantly impacts soil enzyme activities and alters various physicochemical properties of the soil. Both Cd and Pb are toxic to plants even at low concentrations and lack any known biological functions within plant cells (Khellaf and Zerdaoui, 2010).

To address environmental pollution, various remediation technologies based on mechanical or physicochemical principles have been employed to remediate contaminated soil (Marques et al., 2009). These technologies include *in-situ* excavation and landfilling, soil flushing and washing, and electrokinetic methods (Wuana and Okieimen, 2011). However, the aforementioned methods are costly and labor-intensive, disrupt soil structure, reduce indigenous soil microflora, and render the soil unsuitable for plant development (Vangronsveld et al., 2009). Therefore, using plants for soil remediation is considered a biological strategy. Phytoremediation is an effective and cost-efficient method that uses the natural abilities of plants to remove pollutants and reduce hazardous substances in the environment (Carbisu and Alkorta, 2001; Polle et al., 2013). Additionally, it does not adversely affect the chemical quality of the soil (Shrestha et al., 2019). Furthermore, phytoremediation can enhance physical soil quality and improve biological processes (Wan et al., 2016).

A key strategy in phytoextraction is identifying plants that possess tolerance mechanisms against heavy metals and can efficiently uptake them while maintaining significant biomass without adverse impacts on growth (Bhargava et al., 2012; Ye et al., 2017). Plants used in heavy metal phytoremediation should have rapid growth, substantial biomass, and extensive root systems; should be easy and cost-effective to plants; and should have an aesthetically pleasing appearance (Brunner et al., 2008; Luo et al., 2016).

The bioavailability of heavy metals in the soil and their various physiological functions in plant cells (e.g., plant growth stage, soil pH, heavy metal levels, and exposure duration) significantly affect the toxicity, adsorption, and translocation of heavy metals in plants (Bhargava et al., 2012; Zarubova et al., 2015). Additionally, soil physicochemical properties, heavy metal bioavailability, plant and microflora secretions, and the plants’ ability to absorb, accumulate, sequester, or detoxify elements are factors that influence remediation efficiency (Wang et al., 2020).

High levels of ROS caused by heavy metals can damage nucleic acids, peroxidize membrane lipids, oxidize proteins, and inhibit enzymes, ultimately leading to plant cell death. Maintaining a balance between ROS and the antioxidant system is crucial for plants, especially under heavy metal stress (He et al., 2011; Sharma et al., 2012). Plants mitigate oxidative stress through antioxidant enzyme activities [e.g., superoxide dismutase (SOD), glutathione S-transferase (GST), guaiacol peroxidase (POD)] and low molecular weight antioxidants (e.g., ascorbic acid, reduced glutathione, carotenoids, phenolics) (Benavides et al., 2005; Hossain et al., 2012).

Phytochelatins, enzymatically synthesized in plants, are produced in response to heavy metal stress as a defense mechanism (Chia, 2021). Numerous studies have identified several plant species as suitable phytoremediators for Pb- and Cd-contaminated soil, including *Populus* species, *Salix* species (willow), *Jatropha curcas*, and *Schima superba* (Alvarez-Mateos et al., 2019; El-Mahrouk et al., 2020, 2021; Wang et al., 2022). This study focuses on *Conocarpus erectus*, an evergreen tree from the Combretaceae family, as a potential phytoremediator. Indigenous to tropical and subtropical regions, *C. erectus* is extensively used in Egypt for landscaping purposes. Despite its widespread use, its suitability for phytoremediation is poorly understood due to limited data [e.g., growth behavior, phytoremediation efficiency, chemical and biochemical compositions under heavy metals (HMs)] on its remediation capabilities.

Understanding the mechanisms by which plants resist specific metals is necessary to expand the list of plants suitable for treating heavy metal-contaminated soil (Sharma et al., 2023). This study particularly emphasizes Cd and Pb, which are common heavy metals frequently found in soils across various regions of Egypt. The contamination primarily results from the use of sewage sludge and the intensive application of chemical fertilizers, particularly calcium superphosphate, as well as from irrigation water mixed with sewage and industrial effluents. These factors contribute to elevated levels of Cd and Pb in the soil, exacerbating environmental risks.

Given the high mobility of Cd and Pb, which can be magnified through food-web mechanisms, they pose significant threats to living organisms. Therefore, this work was conducted to evaluate the growth, enzymatic activities, electrolyte leakage (EL), and remediation efficacy of *C. erectus* under Cd and Pb stress, individually and in combination.

## 2 Materials and methods

This research was carried out for 16 months, beginning on 1 April 2021 and ending on 1 August 2022 at the Experimental Farm of the Faculty of Agriculture at Kafrelsheikh University in Egypt. The farm is situated at coordinates 31°61′N, 30°57′E with an elevation of 6 m above sea level. Meteorological data from the nearby Sakha meteorological station recorded a total rainfall of 203.28 mm and 111.68 mm, with relative humidity averages of 53.68% and 57.73% and minimum average temperatures of 15.0°C and 16.6°C during the experimental period. Additionally, the average maximum temperatures were recorded as 28.7°C and 29.6°C for the respective seasons.

### 2.1 Plant material

Six-month-old homogeneous transplants of *C. erectus* (36 ± 2 cm ‘height’ and 0.4 cm diameter’ at 3 cm above the soil surface) were obtained from the nursery of the Horticulture Department, Faculty of Agriculture, Kafrelsheikh University and used in this study.

### 2.2 Soil analysis

The soil’s physio-chemical properties were assessed before adding HMs. According to Cools and De Vos (2010), soil samples were ground into particles smaller than 2 mm utilizing a stainless-steel test sieve and pulverized with a mortar and pestle. The particle size distribution was hydrometer-analyzed (Gee and Bauder, 1986). The soil texture was clayey sand (60.05%, 24.03%, 15.92%). The soil pH, electrical conductivity (EC), and organic matter (OM) were 7.68, 1.15 dS/m, and 1.51%, respectively. Nitrogen (N), phosphorus (P), and potassium (K) were 2.80, 3.49, and 221 ppm in the soil. Calcium (Ca^++^), magnesium (Mg^++)^, sodium (Na^+^), and potassium (K^+^) were soil-soluble at 10.00, 7.40, 5.20, and 0.36 mEq/L. Soil chloride (Cl^−^), carbonate (Co_3_^−^), bicarbonate (HCO_3_^−^), and sulfate (SO_4_^−^)were 2.40, 0.00, 1.00, and 19.56 mEq/L. For measuring the soil chemical characteristics, 100 mL of distilled water was added to 20 g of dry soil (1:5, soil:water) for 24 h. The following were determined in the filtered extract: Soil EC was measured according to Jackson (1973) using an EC meter (‘MI 170, ‘SZ egged, ‘Hungary,’ Italy’). For Ca^++^, Mg^++^, and Cl^−^ estimates, the methods of Jackson (1973) were employed. Carbonate volumetrically was determined by a calcimeter and the OM concentration was determined by dichromate oxidation according to Nelson and Sommers (1996). The micro-Kjeldahl approach (Bremner and Mulvaney, 1982) was utilized to assess the available N, whereas the available P was measured according to Olsen et al. (1982). The flame photometer PSP (Jenway, Staffordshire, UK) was utilized to measure Na^+^ and K^+^ (Page et al., 1982). A pH meter (Jenway 3510, Staffordshire, UK) was used to assess soil pH in a suspension (1:2.5, soil:distilled water) after 30 min (Jackson, 1973).

### 2.3 Pollutant treatments

The concentrations of cadmium nitrate [Cd(NO_3_)_2_*4H_2_O] were 40 [low concentration (L)], 60 [medium concentration (M)], and 80 [high concentration (H)] mg kg^−1^ soil, equivalent to 14.4, 21.6, and 28.8 mg Cd kg^−1^ soil, consecutively. On the other hand, the concentrations of lead nitrate [Pb(NO_3_)_2_] were 400 (L), 700 (M), and 1,000 (H) mg kg^−1^ soil, equivalent to 247.70, 433.475, and 619.25 mg Pb kg^−1^ soil, respectively. The plastic pots, 40 cm diameter and 32 cm height, were filled with 9 kg of air-dried soil/pot and mixed with solutions of Cd and Pb at the concentrations above. The plastic pots that contain the polluted soil were placed under a plastic house for 60 days (from 29 January to 31 March) before being cultured. Untreated soil was used as control. The treatments of Cd and Pb were done as follows: control (untreated HM soil), (L) Cd, (M) Cd, (H) Cd, (L) Pb, (M) Pb, (H) Pb, (L) Cd (L) Pb, (M) Cd (M) Pb, and (H) Cd (H) Pb. According to Kabata-Pendias (2011), Cd at 1 to 5 mg kg^−1^ soil and Pb at 20 to 300 mg kg^−1^ soil are the permissible limits around the world.

### 2.4 Planting date

*Conocarpus erectus* transplants (vegetative growth stage) were cultured in plastic pots (one transplant/pot), containing the polluted HM soil on 1 April 2021, and placed in the open field.

### 2.5 The experimental layout

The experiment followed a randomized complete block design, as outlined by Snedecor and Cochran (1989). The experiment consisted of 10 treatments, each replicated three times, with each replication involving three plants, rendering a group of nine plants in each treatment and a total of 90 plants in the experiment.

### 2.6 Agricultural practices

Irrigation was conducted using tap water with a pH of 7.20 and EC of 0.59 dS/m. Furthermore, weed and insect control measures were applied throughout the experimental period.

### 2.7 Recorded data

At the end of the experiment on 1 August 2022, six plants of each treatment (two plants per replicate) were randomly selected to measure the traits described below.

#### 2.7.1 Vegetative characters

The plant height was quantified in centimeters, specifically from the surface of the soil to the highest point of the plant. A portable leaf chlorophyll meter (SPAD-501, Minolta, Osaka, Japan) was utilized to determine the chlorophyll index, with the apex meristem of the fifth leaf selected (Markwell et al., 1995). Additionally, the measurements of the longest root (in centimeters) and the dry weights (in grams) of the leaves, stems, and roots were recorded. The harvested plants were divided into leaves, stems, and roots. These plant parts were subsequently rinsed with tap water to eliminate soil and further purified using deionized water. Afterward, the different sections of the plant were dehydrated in an oven at a temperature of 80°C for a duration of 24 h (Rautio et al., 2010).

#### 2.7.2 Assays of antioxidant enzyme activities

In order to evaluate antioxidant enzyme activities (AEAs), 0.5g of completely developed young leaves (the fifth leaf from the top of the plant) were crushed in liquid nitrogen together with 3 mL of extraction buffer. The extraction buffer consisted of 50 mM of TRIS buffer (pH 7.8), 1 mM of EDTA-Na_2_, and 7.5% polyvinylpyrrolidone. The crushing process was carried out using a mortar and pestle that had been precooled. Subsequently, the resulting mixture was passed through four layers of cheesecloth and centrifuged at 12,000 rpm for 20 min at 4°C. The supernatant obtained was subjected to secondary centrifugation under identical conditions and then utilized to assess the activity of total soluble enzymes via an ultraviolet 160 A spectrophotometer (Shimadzu, Japan).

For CAT estimation, the H_2_O_2_ consumption at 240 nm was monitored, following the method outlined by Aebi (1984). The reaction mixture comprised 20 mg of total protein, 50 mM of sodium phosphate buffer (pH 7.0), and 10 mM of H_2_O_2_. Catalase activity was quantified as a 0.01 reduction in absorbance at 240 nm per mg of protein per minute.

The activity of ‘PPO’ was measured following the methodology delineated by the protocol of Malik and Singh (1980). The reaction mixture consisted of 3.0 mL of buffered catechol (0.01 M) and 0.1 M of phosphate buffer (pH 6.0). At 30 s and 3 min after adding 100 μL of crude enzyme extract, variations in absorbance at 495 nm were noted. The rise in absorbance per minute per gram of fresh weight was used to express PPO activity.

For POD activity determination, the protocol by Hammerschmidt et al. (1982) was followed with some modifications. The ‘reaction’ mixture was composed of 2.9 mL of 100 mM sodium phosphate buffer (pH 6.0) containing 0.25% (V/V) ‘guaiacol’ and 100 mM of H_2_O_2_. Over the course of 3 min, variations in absorbance at 470 nm were monitored at 30-s intervals following the addition of 100 μL of crude enzyme extract. The increase in absorbance per minute per gram of fresh weight constituted POD activity.

#### 2.7.3 Electrolyte leakage

EL was performed according to Szalai et al. (1996), with some modifications. Twenty discs of leaves (1 cm²/disc) had been placed singly into flasks with 25 mL of distilled water (Milli-Q50, Millipore, Bedford, MA). To facilitate EL from injured tissues at an ambient temperature, a flask was shaken for 20 h. The EC was determined by registering each vial using an Acro met AR20 EC meter (Fisher Scientific, Chicago, IL). The flasks were immersed in a hot water bath (Fisher Isotemp, Indiana, PA) at a temperature of 80°C (176°F) for 1 h in order to cause the cell to rupture. The vials were once again positioned on the Innova 2100 platform shaker for a duration of 20 h at a temperature of 21°C (70°F). Final conductivity had been recorded per flask. EL% per baud was recorded as the first conductivity/end conductivity × 100.

#### 2.7.4 Cadmium and lead concentrations in plant organs

A metal-free mill (IKA-Werke, M20 Germany) was used to ground the dried samples of leaves, stems, and roots to obtain a homogeneous power. Five milliliters of concentrated sulfuric acid (95%) was carefully dropped on the sample (0.2 g), and then a sand hotplate was used to heat the mixture for 10 min. After that, perchloric acid (0.5 mL) was added to obtain a clear solution, and the heating was continued. The solution was filtered after cooling and diluted to 50 mL with deionized water (Evenhuis and de Waard, 1980). Cd and Pb contents (mg kg^−1^ dry weight) were measured according to Page et al. (1982) in the leaves, stems, and roots of the plant by atomic absorption spectrophotometry (Avanta E; GBC).

#### 2.7.5 Bioconcentration factor, translocation factor, and tolerance index

To evaluate the remediation ability of *C. erectus* for Cd and Pb and its tolerance against Cd and Pb. Bioconcentration factor (BCF), translocation factor (TF), tolerance index (TI) biomass (TI_b_), and TI roots (TI_r_) were calculated as follows:


$$\text{BCF}=\frac{\text{Metal concentration in plant part }\left(\text{mg}/\text{kg D}.\text{W}\right)}{\text{Soil metal level }\left(\text{mg}/\text{kg soil D}.\text{W}.\right)\;}$$

According to BCF values, the accumulation potential was evaluated and classified as follows: intensive, BCF > 1; medium, BCF = 1–0.1; weak, BCF = 0.1–0.01; and no accumulation, BCF = 0.01–0.001 (Kabata-Pendias and Pendias, 1999).


$$\text{TF}=\frac{\text{The shoots metal concentration}\left(\text{mg }/\text{kg D}.\text{W}.\right)\;}{\text{The root metal concentration}\left(\text{mg }/\text{kg D}.\text{W}.\right)\;}$$

The TF was calculated to evaluate the transfer of metal ions from the roots to the aboveground portions of the plant (Maiti and Jaiswal, 2008), specifically including the shoots, which comprise both the leaves and stems.


$$\text{TIb}=\frac{\text{Metal treated plant D}.\text{W }\left(\text{g}/\text{ plant}\right)}{\text{Control plant D}.\text{W }\left(\text{g}/\text{plant}\right)}$$

Wilkins (1978) proposed a method for assessing the tolerance of plants to HMs; this method involves calculating TI_b_ based on the following three values: TI_b_ < 1: this indicates a net reduction in plant weight, suggesting that the plants are under stress due to heavy metal exposure; TI_b_ = 1: this implies that there is no difference in plant weight compared to the control treatment (no heavy metal exposure); and TI_b_ > 1: this indicates a net increase in plant weight and healthy growth, suggesting that the plant is tolerating the heavy metals effectively.

Also, TI_r_ has been determined by Wilkins (1978), where:


$$\text{TIr}=\frac{\text{Length of longest root in the metal treated plant }\left(\text{cm}\right)}{\text{Length of longest root in control plant }\left(\text{cm}\right)\;}$$

### 2.8 Statistical analysis

The results were analyzed using the SAS program (Version 6.12; SAS Institute Inc., Cary, NC, USA). Mean separation (± SE) was determined using Duncan’s multiple range test in a one-way ANOVA, with significance level estimated at *p ≤*0.05. The correlation analysis among the measured parameters was conducted using SPSS software, version 28.

## 3 Results

### 3.1 Growth traits and tolerance index

**Figure 1:**
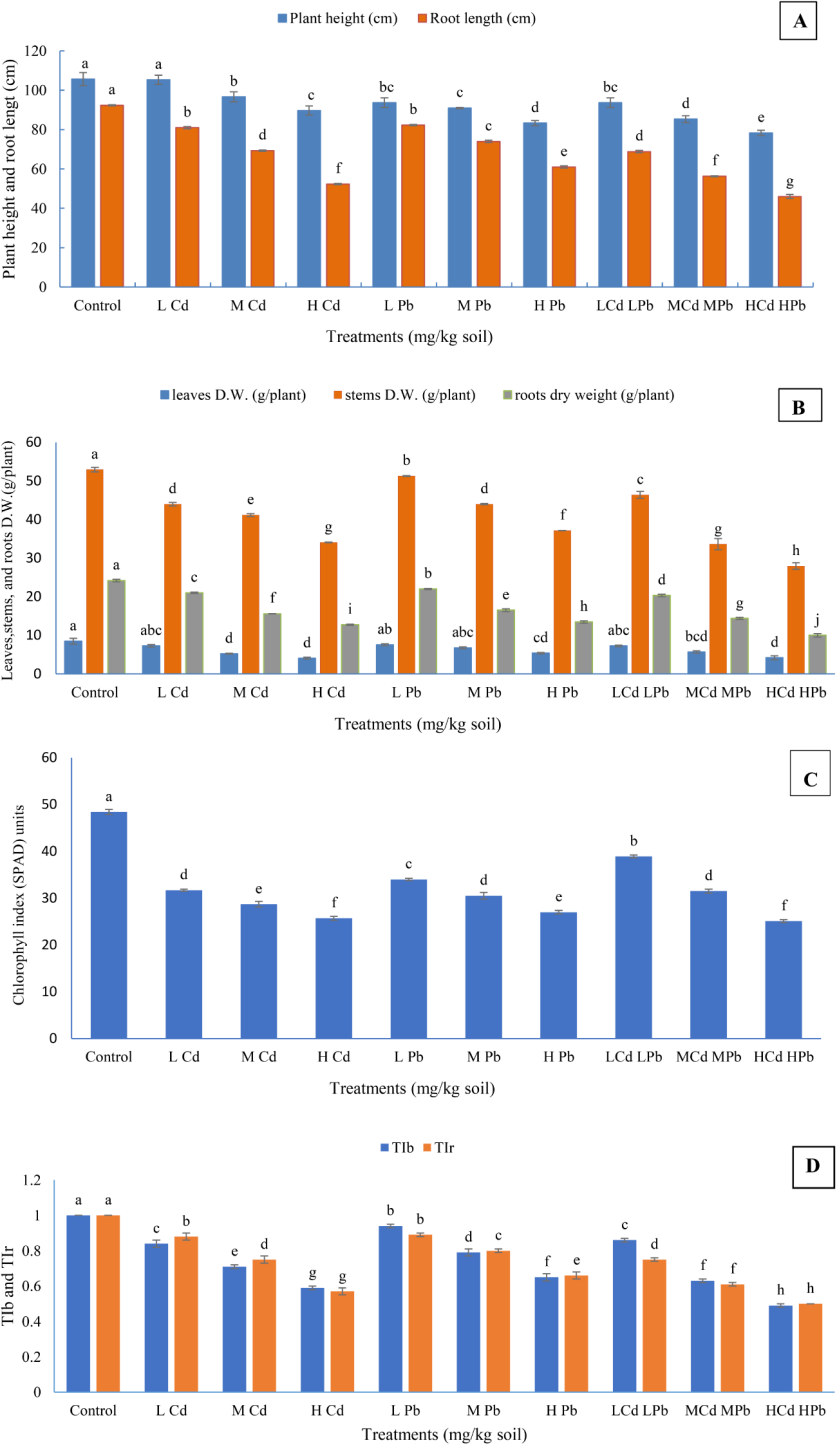
Effect of Cd and Pb treatments on plant height, root length **(A)**; dry weights of the leaves, stems, and roots **(B)**; chlorophyll index of *Conocarpus erectus* **(C)**; and tolerance index biomass (TI_b_) and tolerance index root (TI_r_) **(D)**. The data are presented as the mean ± S.E. The same letters in the figure denote no significance among the mean values by Duncan’s multiple range test (*p* ≤ 0.05).

The application of various levels of Cd and Pb, either individually or in combinations, had significant negative effects on the growth traits (**Figures 1A–C**). The reductions in plant height, chlorophyll index, and dry weights of the leaves, stems, and roots per plant, as well as root length, were significant compared to the control. Exceptions included treatments with (L) Cd (in case of plant height), as well as (L) Cd, (L) Pb, (M) Pb, and (L) Cd (L) Pb treatments (in case of leaves dry weight/plant), which showed non-significant reduction in comparison to the control. The decrease in growth trait values was relative to the increase in Cd or Pb levels in the soil. It was also noticed that Cd negatively affected the growth traits more than Pb when they were singly applied. Moreover, the growth trait values were gradually decreased with an increase in the combination levels of Cd and Pb. The highest significant values for the plant height; chlorophyll index; dry weights of the leaves, stems, and roots; and root length were observed in control plants: 105.67 cm, 48.40 SPAD units, 8.43 g, 52.93 g, 24.17 g per plant, and 92.33 cm, respectively. In contrast, significantly lower values for the aforementioned traits were recorded for the plants treated with high concentrations of Cd and Pb, with measurements of 78.33 cm, 24.70 SPAD units, 4.16 g, 27.92 g, 9.99 g per plant, and 46.00 cm, respectively. Furthermore, both the growth characteristics and the levels of Cd and Pb in the applied treatments were crucial factors for their impact on the growth traits. For instance, the application of (H) Cd treatment resulted in a reduction of 15.06%, 46.16%, 52.19%, 35.63%, 47.37%, and 42.68% in plant height; chlorophyll index; dry weights of the leaves, stems, and roots per plant; and root length, respectively, compared to the control. Similarly, the application of (H) Pb treatment led to reductions of 21.14%, 42.91%, 36.42%, 29.89%, 44.44%, and 33.93% in these traits, respectively, relative to the control. Moreover, the combined treatment of (H) Cd and (H) Pb resulted in sequential reductions of 25.87%, 48.97%, 50.65%, 47.25%, 58.67%, and 50.18% in these growth parameters, respectively. The data clearly indicate that the deleterious effects of Cd and Pb on growth traits follow the order of Cd + Pb > Cd > Pb in most cases. In general, significant differences among the applied treatments were observed for the majority of growth parameters (*p* ≤ 0.05).

During the experimental period, harmful visual effects such as leaf chlorosis and drying of the leaf edges were observed on mature leaves, especially under high Cd and Pb concentrations, either individually or in combination. The visual toxicity is reduced by increasing the growth stage. The TI of either biomass or roots gradually decreases as the concentrations of Cd or Pb in the soil increase. *Conocarpus erectus* had a good tolerance mechanism against the applied HM treatments, particularly under low and medium levels, with 100% survival until the end of the experimental time. *Conocarpus erectus* performed a better resistance over 70% for TI_b_ or TI_r_ under the lower and medium concentrations of Cd and Pb applied singly or (L) Cd (L) Pb (**Figure 1D**). The plants have performed the least tolerance under (H) Cd (H) Pb treatment, where TI_b_ and TI_r_ under such a treatment reached 0.49 (49%) and 0.50 (50%), respectively. Regardless of the control, the highest significant TI_b_ and TI_r_ were 0.94 and 0.89 (94% and 89%) under (L) Pb treatment, respectively. Under the other treatments, the TI_b_ and TI_r_ values were intermediate and showed significant differences between each other.

### 3.2 Enzyme activities

**Figure 2:**
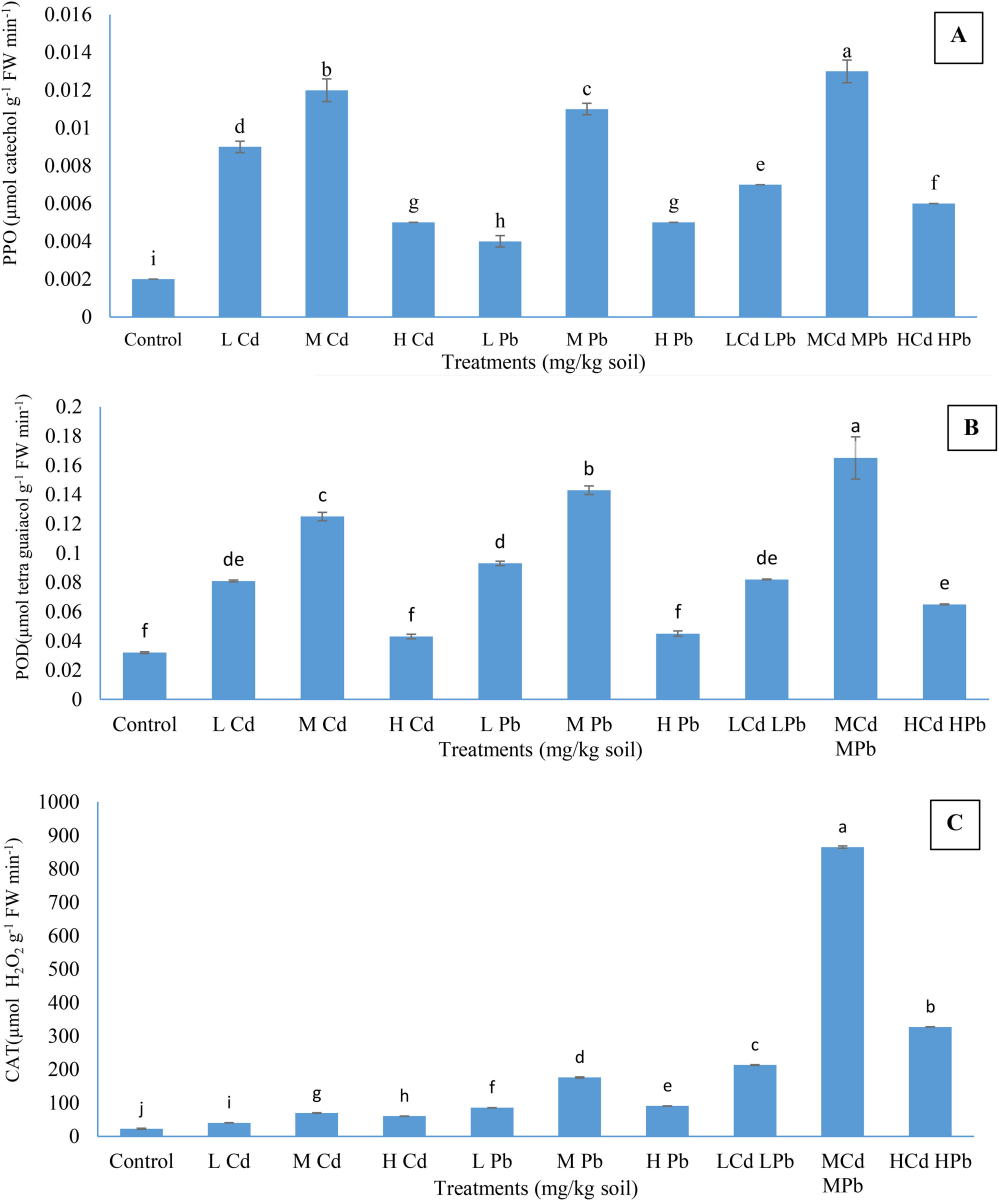
Effect of Cd and Pb treatments on polyphenol oxidase (PPO) **(A)**, peroxidase (POD) **(B)**, and catalase (CAT) **(C)** activities in *Conocarpus erectus* leaves. The data are presented as the mean ± S.E. The same letters in the figure denote no significance among the mean values by Duncan’s multiple range test (*p* ≤ 0.05).

The data presented in **Figures 2A–C** indicate significantly higher activities of PPO, POD, and CAT in the leaves of plants cultivated in soil with varying concentrations of Cd and Pb, both individually and in combination, compared to the respective control plants. It was observed that the enzyme activities have been raised with the upheaval of Cd and Pb levels, whether applied individually or in combination, until reaching medium concentrations; thereafter, a decline was noted at higher concentrations of heavy metals, albeit remained elevated compared to the control plants. The results demonstrated that (M) Cd (M) Pb treatment induced significantly higher enzyme activities compared to the other treatments. Specifically, this treatment resulted in the activation of PPO, POD, and CAT to levels of 0.013 µmol catechol min^−1^ g^−1^ fresh weight (FW), 0.165 µmol tetraguaiacol g^−1^¹ FW min^−1^¹, and 865.05 µmol H_2_O_2_ g^−1^ FW min^−1^, respectively. Meanwhile, treatments with (M) Cd or (M) Pb resulted in PPO activities of 0.012 and 0.011 µmol catechol g^−1^ FW min^−1^, POD activities of 0.0125 and 0.0143 µmol tetraguaiacol g^−1^ FW min^−1^, and CAT activities of 69.54 and 176.05 µmol H_2_O_2_ g^−1^ FW min^−1^, respectively. In contrast, the activities of PPO, POD, and CAT in the control plants were measured at 0.002 µmol catechol g^−1^ FW min^−1^, 0.032 µmol tetraguaiacol g^−1^ FW min^−1^, and 22.65 µmol H_2_O_2_ g^−1^ FW min^−1^, respectively.

### 3.3 Electrolyte leakage

**Figure 3:**
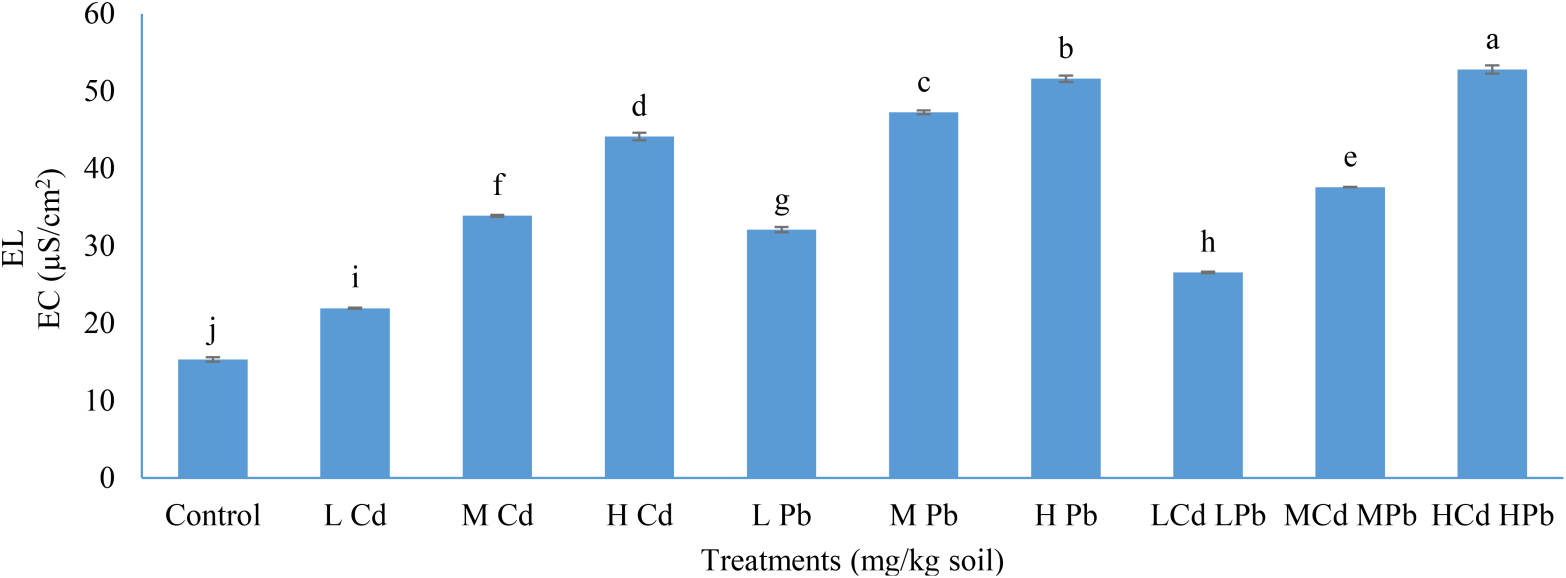
Effect of Cd and Pb applications on electrolyte leakage (EL) in *Conocarpus erectus*. The data are presented as the mean ± S.E. The same letters in the figure denote no significance among the mean values by Duncan’s multiple range test (*p* ≤ 0.05).

Regarding the results of EL, it was observed that the value of EL significantly upheaved with the rise in the soil levels of Cd and Pb, individually or in combination (**Figure 3**).

Overall, the most substantial increase in EL was observed with the application of (H) Cd (H) Pb, resulting in a 52.79% elevation, followed by (H) Pb with a 51.60% increase, and (H) Cd with a 44.15% rise, compared to the 15.33% EL recorded in the control plants. Furthermore, all applications involving Cd and Pb demonstrated statistically significant elevations (*p* ≤ 0.05) in EL when compared to each other. Additionally, the combined presence of Cd and Pb exerts a more pronounced detrimental effect on cell membrane permeability than their individual presence in the soil. The applications of (L) Cd, (M) Cd, (L) Pb, (M) Pb, (L) Cd (L) Pb, and (M) Cd (M) Pb resulted in 21.95, 33.90, 32.10, 47.28, 26.59, and 37.60 EC (µS/cm^2^), respectively.

### 3.4 Influence of Cd and Pb levels in the soil on their contents in the plant organs

**Figure 4:**
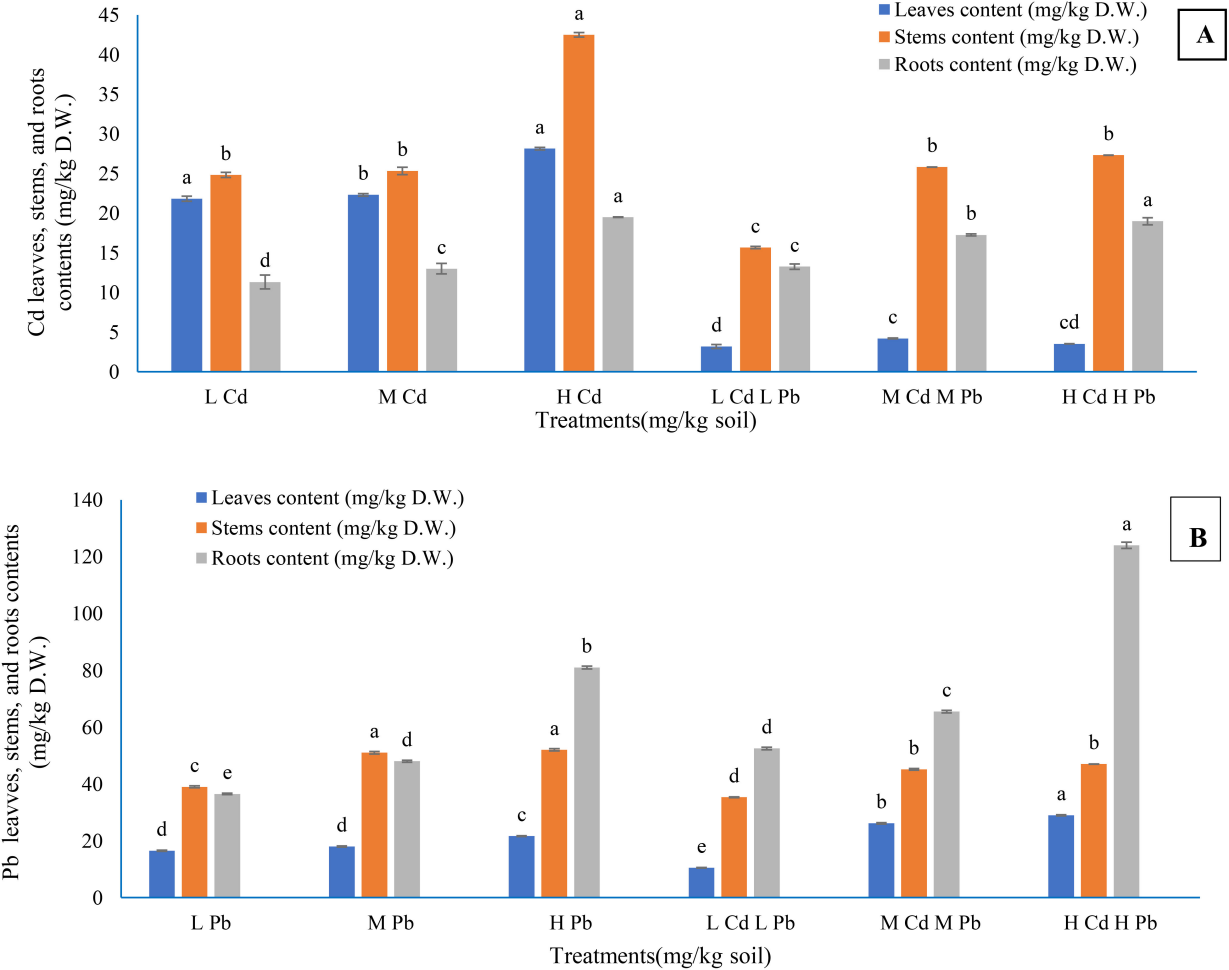
The contents of cadmium **(A)** and lead **(B)** in *Conocarpus erectus* leaf, stem, and root as affected by soil Cd and Pb levels. The data are presented as the mean ± S.E. The same letters in the figure denote no significance among the mean values by Duncan’s multiple range test (*p* ≤ 0.05).

The findings presented in **Figures 4A, B** elucidate that the concentrations of both Cd and Pb in plant parts have escalated with the elevation of their respective concentrations in the soil, whether applied individually or in combination. The Cd concentrations in the leaves and stems when Cd was added singly were higher than their concentrations under the combination of the two metals at the same level. The contract happened in the root Cd concentration. Pb concentration in the leaves, stems, and roots took the same manner as Cd except for the leaf Pb concentration under (M) Cd (M) Pb and (H) Cd (H) Pb treatments, which was higher than its concentrations under (M) Pb and (H) Pb treatments. Higher and significant Cd concentrations reached 28.16, 42.50, and 19.50 mg/kg D.W. in the leaves, stems, and roots, respectively, as a result of (H) Cd treatment in addition to (H) CD (H) Pb treatment in the case of roots (19.00 mg/kg D.W.). In contrast, (H) Cd (H) Pb treatment achieved higher and significant leaf and root Pb contents of 29.00 and 124.00 mg/kg D.W., respectively. Conversely, the highest significant stem Pb content (52.00 mg/kg D.W.) resulted from (H) Pb treatment. The distribution of Cd content in the plant organs under the treatments containing Cd only followed the order of stems > leaves > roots, while under the treatments containing Cd combined with Pb exhibited the order of stems > roots >leaves. Furthermore, the content of Pb in the plant components followed the order of roots > stems > leaves, except for Pb content observed under (L) Pb and (M) Pb treatments, which followed the order of stems > roots > leaves.

### 3.5 Evaluation of *Conocarpus erectus* phytoremediation potential

Bioconcentration factors of the shoots (BCF_s_) and roots (BCF_r_), as well as TF, were calculated to estimate the phytoremediation efficacy of *C. erectus* under various Cd and Pb concentrations (**Table 1**). In general, BCF_s_, or BCF_r_ of Cd and Pb, are dependent on the soil metal level and their presence individually or in combination. It is observed that the BCF_s_ of Cd or Pb were higher under their addition individually than in combination. Additionally, when the metal level in the soil increased, the BCF_s_ of the metal decreased. In contrast, the BCF_r_ of Cd and Pb under their combination treatments were higher than those observed under single metal additions. According to Kabata-Pendias and Pendias (1999), Cd BCF_s_ >1 and Cd BCF_r_ <1 are estimated as intensive and medium, respectively, while Pb BCF_s_ and BCF_r_ = (1–0.1) are considered as medium. The highest significant Cd BCF_s_ and BCF_r_ were 3.24 and 0.92 calculated for the treatments of (L) Cd and (L) Cd (L) Pb, consecutively. On the other hand, Pb BCF_s_ and BCF_r_ recorded the highest of 0.23 and 0.21 under (L) Pb and (L) Cd (L) Pb applications, respectively. Conversely, the (H) Cd (H) Pb treatment induced a lower significant Cd BCF_s_ (1.07) and Pb BCF_s_ (0.12), in addition to the (H) Pb treatment, which also recorded a Pb BCF_s_ of 0.12. Meanwhile, the plants grown in (M) Cd and (M) Pb recorded the least significant BCF_r_ of Cd (0.60) and BCF_r_ of Pb (0.10).

**Table 1:** Bioconcentration factors of the shoots (BCF_s_) and roots (BCF_r_) and translocation factor (TF) of Cd and Pb of *Conocarpus erectus* as impacted with soil Cd and Pb levels.

Treatments (mg/kg soil)	BCF_s_	BCF_r_	TF
Cd			
(L) Cd	3.24 ± 0.06 a	0.78 ± 0.02 b	4.12 ± 0.08 a
(M) Cd	2.21 ± 0.03 c	0.60 ± 0.02 d	3.68 ± 0.18 b
(H) Cd	2.45 ± 0.05 b	0.68 ± 0.01 c	3.63 ± 0.13 b
(L) Cd (L) Pb	1.31 ± 0.04 d	0.92 ± 0.01 a	1.42 ± 0.04 c
(M) Cd (M) Pb	1.39 ± 0.06 d	0.80 ± 0.03 b	1.74 ± 0.01 c
(H) Cd (H) Pb	1.07 ± 0.05 e	0.66 ± 0.00 cd	1.62 ± 0.07 c
Pb			
(L) Pb	0.23 ± 0.00 a	0.15 ± 0.00 b	1.53 ± 0.07 a
(M) Pb	0.14 ± 0.00 c	0.10 ± 0.00 d	1.44 ± 0.01 a
(H) Pb	0.12 ± 0.00 d	0.13 ± 0.00 c	0.91 ± 0.00 c
(L) Cd (L) Pb	0.18 ± 0.00 b	0.21 ± 0.00 a	0.88 ± 0.05 c
(M Cd (M) Pb	0.15 ± 0.00 c	0.14 ± 0.00 bc	1.09 ± 0.03 b
(H) Cd (H) Pb	0.12 ± 0.00 d	0.20 ± 0.00 a	0.62 ± 0.02d

The data are presented as the mean ± S.E. The same letters in the table denote no significance among the mean values by Duncan’s multiple range test (p ≤ 0.05).

Concerning the results of TF, which determine the transportation of metal quantity from the roots to aerial plant organs, the results indicated that the TF of Cd or Pb values reduced when each was raised in the treatments with two exceptions: TF Cd and TF Pb in (M) Cd (M) Pb. The translocation factor of Cd was >1 (>100) in all utilized applications, and the highest and least significant TF values of Cd were 4.12 (412%) and 1.42 for the plants grown in (L) Cd and (L) Cd (L) Pb applications, respectively.

Notably, Pb migration from the roots to aboveground plant organs surpassed unity (>100%) under (L) Pb, (M) Pb, and (M) Cd (M) Pb treatments reaching 1.53, 1.44, and 1.09, respectively. Conversely, under (H) Pb, (L) Cd (L) Pb, and (H) Cd (H) Pb treatments, the Pb migration from the roots to aerial parts was limited, as the TF values for Pb were <1 (<100%), reaching 0.91, 0.88, and 0.62, consecutively.

### 3.6 The correlation between the measured traits

The data presented in **Table 2** demonstrate a highly significant positive correlation between vegetative traits (including plant height, chlorophyll index, and dry weights of the leaves, stems, and roots) and the tolerance index of biomass and roots. Conversely, PPO showed a negative, albeit not statistically significant, correlation with most vegetative traits (excluding chlorophyll index, where the correlation is both negative and statistically significant), as well as with TI_b_ and TI_r_. However, PPO exhibited significantly positive correlations with POD, CAT, and EL. Moreover, POD displayed a negative, albeit not statistically significant, correlation with most vegetative traits (excluding dry weight of the leaves) as well as with TI_b_ and TI_r_. However, the correlation is positive, though not statistically significant, with the dry weight of the leaves and EL. The correlation between POD and CAT, however, is highly significant (*p* ≤ 0.01). CAT demonstrated a significant negative correlation with plant height, root dry weight, and TI_b_ and a non-significant negative correlation with chlorophyll index and leaf dry weight while showing a non-significant positive correlation with EL. Notably, CAT exhibited a highly significant negative correlation with stem dry weight, root length, and TI_r_. Finally, EL showed a significant negative correlation with vegetative traits, TI_b_, and TI_r_.

**Table 2:** The correlations between vegetative traits, tolerance index of biomass (TI_b_) and roots (TI_r_), enzyme types, and electrolyte leakage (EL).

Traits	Plant height	Chlorophyll index	Leaves D.W.	Stems D.W.	Roots D.W.	Length of root	TI_b_	TI_r_	PPO	POD	CAT	EL
**Plant height**	1^n.s.^											
**Chlorophyll index**	0.475^**^	1^n.s.^										
**Leaves D.W.**	0.550^**^	0.848^**^	1^n.s.^									
**Stems D.W.**	0.546^**^	0.804^**^	0.896^**^	1^n.s.^								
**Roots D.W.**	0.593^**^	0.853^**^	0.940^**^	0.943^**^	1^n.s.^							
**Length of root**	0.573^**^	0.773^**^	0.898^**^	0.940^**^	0.938^**^	1^n.s.^						
**TI_b_**	0.557^**^	0.841^**^	0.944^**^	0.977^**^	0.977^**^	0.951^**^	1^n.s.^					
**TI_r_**	0.595^**^	0.780^**^	0.898^**^	0.936^**^	0.937^**^	0.989^**^	0.947^**^	1^n.s.^				
**PPO**	−0.127^n.s.^	−0.381^*^	−0.240^n.s.^	−0.331^n.s.^	−0.313^n.s.^	−0.251^n.s.^	−0.314^n.s.^	−0.249^n.s.^	1 ^n.s.^			
**POD**	−0.135^n.s.^	−0.213^n.s.^	0.005^n.s.^	−0.085^n.s.^	−0.105^n.s.^	−0.063^n.s.^	−0.079^n.s.^	−0.068^n.s.^	0.854^**^	1^n.s.^		
**CAT**	−0.391^*^	−0.185^n.s.^	−0.234^n.s.^	−0.482^**^	−0.370^*^	−0.480^**^	−0.413^*^	−0.477^**^	0.549^**^	0.614^**^	1^n.s.^	
**EL**	−0.640^**^	−0.812^**^	−0.767^**^	−0.742^**^	−0.887^**^	−0.782^**^	−0.802^**^	−0.783^**^	0.161^n.s.^	0.103^n.s.^	0.228^n.s.^	1^n.s.^

N = 30; *p < 0.05; **p < 0.01; n.s., non-significant.

Regarding the correlation among Cd parameters, the data in **Table 3** indicate notable patterns. Specifically, Cd leaf concentration exhibits a highly significant positive correlation with Cd stem concentration, Cd bioconcentration factors (BCF_s_), and Cd translocation factor (TF). Conversely, its correlation with Cd root concentration and Cd BCF_r_ is non-significant and significantly negative, respectively. Moreover, the correlation between Cd stems and Cd roots is highly significant and positive, while it is significantly negative with Cd BCF_s_ and Cd BCF_r_ and non-significant with Cd TF. Similarly, the correlation between Cd roots and Cd BCF_s_, BCF_r_, and TF is non-significant and negative. Furthermore, Cd BCF_s_ demonstrate a non-significant negative correlation with Cd BCF_r_ and Cd TF, while it exhibits a highly significant positive correlation with Cd TF. Conversely, Cd BCF_r_ correlates negatively and non-significantly with Cd TF.

**Table 3:** The correlations between Cd parameters.

Traits	Concentration in the leaves	Concentration in the stems	Concentration in the roots	BCF_s_	BCF_r_	TF
**Concentration in the leaves**	1^n.s.^					
**Concentration in the stems**	0.630**	1^n.s.^				
**Concentration in the roots**	−0.133^n.s.^	0.649^**^	1^n.s.^			
**BCF_s_**	0.856^**^	−0.346^n.s.^	−0.444^n.s.^	1^n.s.^		
**BCF_r_**	−0.495^*^	−0.538^*^	−0.267^n.s.^	−0.167^n.s.^	1^n.s.^	
**TF**	0.946**	0.466^n.s.^	−0.347^n.s.^	0.940^**^	−0.465^n.s.^	1^n.s.^

N = 18; *p < 0.05; **p < 0.01; n.s., non-significant.

Regarding the correlation between Pb parameters as depicted in **Table 4**, it is evident that Pb leaf concentration exhibits significant positive correlations with Pb concentrations in the stems and roots, each being highly significant. Additionally, the correlation between Pb leaf concentration and each of Pb’s BCF_s_, BCF_r_, and TF is notably negative and highly significant. Conversely, the correlation between Pb stem concentration and Pb root concentration is non-significant and significantly negative with Pb’s BCF_s_ and BCF_r_, while it remains non-significant with Pb TF. Moreover, the correlation between Pb root concentration and Pb’s BCF_s_, BCF_r_, and TF is highly significant, non-significant, and highly significant, respectively. Furthermore, Pb BCF_s_ demonstrates a non-significant positive correlation with both Pb BCF_r_ and Pb TF, successively, while Pb BCF_r_ exhibits a notably negative and highly significant correlation with Pb TF.

**Table 4:** The correlations between Pb parameters.

Traits	Concentration in the leaves	Concentration in the stems	Concentration in the roots	BCF_s_	BCF_r_	TF
**Concentration in the leaves**	1^n.s.^					
**Concentration in the stems**	0.589^*^	1^n.s.^				
**Concentration in the roots**	0.748**	0.392^n.s.^	1^n.s.^			
**BCF_s_**	−0.592^**^	−0.743^**^	−0.713^**^	1^n.s.^		
**BCF_r_**	−0.081^n.s.^	−0.654^**^	0.403^n.s.^	0.143^n.s.^	1^n.s.^	
**TF**	−0.377^n.s.^	−0.029^n.s.^	−0.83^**^	0.602^**^	−0.677^**^	1^n.s.^

N = 18; *p < 0.05; **p < 0.01; n.s., non-significant.

## 4 Discussion

Agricultural soil polluted with HMs is a critical issue that poses environmental hazards due to its adverse impacts on various ecological aspects (Masakorala et al., 2013). Certain plants possess significant potential for remediating contaminated soil by absorbing HMs and storing them in their aerial organs (phytoextraction), consequently reducing the bioavailability of pollutants in the soil (Nwoko, 2010). Kaur et al. (2024) observed that the accumulation of Cd and Pb in plants disrupted functional groups such as carboxyl, amino, hydroxyl, and phosphate, leading to unintended alterations in physiological and biochemical processes. For instance, these metals disrupt photosynthesis by impacting chloroplast structure and function, as observed with Cd exposure (Jing et al., 2005), resulting in diminished chlorophyll content. Consequently, the plant’s capacity to harness energy via photosynthesis is compromised, leading to stunted vegetative growth. Furthermore, heavy metals like Pb can induce the generation of ROS, inducing oxidative stress in plants (Kupper, 2017). Pb or Cd can disrupt the uptake and translocation of water and essential nutrients in plants, leading to imbalances and hindering root growth. This disruption can result in decreased growth, abnormalities in phloem and xylem structure, and impairment of biochemical and physiological processes (Kumar et al., 2017; Wu et al., 2018). Furthermore, these metals can modulate the expression of genes associated with growth and development, leading to alterations in hormone production and other signaling molecules that regulate plant growth (Alaraidh et al., 2018). Consequently, stunted vegetative growth and reduced chlorophyll content may occur.

In the current study, the vegetative measurements (**Figures 1A, B**) are consistent with several investigations that have documented the negative impacts of Cd and Pb at various concentrations on vegetative traits. For instance, Tauqeer et al. (2016) observed a significant retardation in the root dry weight of *Alternanthera bettzickiana* at 0.225 mg Cd/L or Pb at 0.414 mg/L. Similarly, Redovnikovic et al. (2017) found that the dry weights of aerial parts and roots of *Populus nigra* cv. ‘Italica’ were negatively affected by various combinations of Pb at 400–1,200 and Cd at 10–50 mg/kg soil, but the Cd and Pb combination at a higher rate had so much negative effect. Other studies on various plant species such as *P. nigra* (El-Mahrouk et al., 2020), *Clidemia sericea* (Durante-Yanez et al., 2022), *S. superba*, Chinese sweetgum, and Chinese fir (Wang et al., 2022) have also reported the detrimental effects of Cd and Pb. Furthermore, Yousefi et al. (2023) observed that elevated Cd levels resulted in reduced plant height and diminished growth of *Portulaca oleracea*. Consistent with these results, Saleem et al. (2024) observed that exposure to 50 and 100 µM of Cd resulted in reduced plant growth and chlorophyll content in *Pisum sativum*.

The findings of Kumar et al. (2023) and Li et al. (2023) underscore the impact of Cd and Pb toxicity on various aspects of plant physiology, including the inhibition of seedling growth, the synthesis of photosynthetic pigments, and the disruption of biological and physiological activities. This reduction in chlorophyll pigment content under HM stress can be attributed to the inhibition of enzymes responsible for their biosynthesis and the substitution of Mg^2+^ in the chlorophyll molecule by HMs, leading to diminished chlorophyll levels and compromised photosynthetic efficiency (Rastgoo and Alemzadeh, 2011; Sumalan et al., 2023), along with degradation of chloroplast architecture, disruption of photosynthetic enzyme activity, and stomatal closure (Chandra and Kang, 2015). All the aforementioned research aligns with our findings of chlorophyll index (**Figure 1C**). However, our research on *C. erectus* indicates that it can thrive under conditions of Cd and Pb stress. Similarly, Kaur et al. (2024) found that *Salix alba* exhibited a survival rate of over 85% when grown in soil with Pb concentrations of 200 mg/kg and Cd concentrations of 15 mg/kg.

According to Wilkins (1978), a TI_b_ or TI_r_ value of less than 1 indicates that a plant is under stress, characterized by a decrease in weight and significant depletion of root growth. This observation aligns with our findings (**Figure 1D**). The level of tolerance exhibited by the plants is contingent upon the specific plant species or clones. Similarly, Dos Santos Utmazian et al. (2007) observed variations in the tolerance levels of 20 different willow and poplar clones when exposed to Cd and Zn. Moreover, the TI_b_ and TI_r_ of *P. nigra* were <1 at Cd Cl_2_ (20, 40, 60, and 80) and Pb acetate (250, 450, 650, and 850) mg/kg soil (El-Mahrouk et al., 2020). Using lower levels of Cd and Pb, *C. erectus* showed excellence in tolerance mechanism, reaching over 80% and 90% against Cd and Pb, respectively, and it had a good defense against Cd and Pb at medium levels.

ROS are generated due to the incomplete reduction of molecular oxygen, resulting in the formation of hydroxyl radicals (OH), hydrogen peroxide (H_2_O_2_), superoxide anions (O^2−^), and ozone (O_3_) (Nathan and Ding, 2010). While ROS are essential for signaling pathways and are naturally produced in cells, adverse conditions cause higher plants to generate more ROS, leading to a wide range of physiological changes. Elevated ROS levels can degrade antioxidants, triggering the expression of antioxidative response genes (Mansoor et al., 2023). However, excessive ROS inflict damage on cellular components such as proteins, lipids, and DNA; disrupt normal mitosis; and ultimately culminate in senescence and cell death (Santos et al., 2010; Kupper, 2017; Bashir et al., 2021; Hendrix et al., 2023). To combat ROS, plants possess a sophisticated antioxidative defense system comprising both non-enzymatic and enzymatic components, which serve as a natural defense mechanism against increased concentrations of heavy metals (Li et al., 2023). Enzymatic components such as PPO, POD, and CAT play a crucial role in preventing the overproduction of ROS and subsequent oxidative damage due to heavy metal exposure, thereby safeguarding the plant from oxidative harm (Yang et al., 2009; Sofy et al., 2020). PPO is a terminal oxidase that facilitates the direct transfer of electrons to O_2_ during the oxidation of intermediate products in plant respiration. It catalyzes the transformation of compounds like phenol into quinone and is involved in the synthesis of phenol-containing cell wall compounds such as lignin. Under heavy metal stress, PPO activity increases significantly, aiding in plant detoxification by converting phenolic compounds into less reactive forms and thereby reducing ROS accumulation (Rivero et al., 2001). Supporting this notion, Battana and Ghanata (2014) demonstrated in their study on *pigeon pea* that Cd detoxification occurs through the formation and sequestration of Cd–Ca crystals during phenol polymerization by phenol oxidases. Another category of enzymes involved in the breakdown of hydrogen peroxide (H_2_O_2_) is POD, which converts H_2_O_2_ to water by oxidizing various substrates simultaneously (Nianiou-Obeidat et al., 2017). POD exists in numerous isoforms and participates in a wide array of cellular activities, such as growth and development, differentiation, lignification, senescence, and auxin degradation, utilizing a diverse range of phenolic substrates (Passardi et al., 2004). Additionally, CAT is a “heme-containing” enzyme and plays a crucial role in scavenging H_2_O_2_ (Sharma et al., 2012). CAT directly degrades H_2_O_2_ without the need for an electron donor, resulting in the formation of H_2_O + O_2_ (Ahmad et al., 2010). The observed delay in the enhancement of photosynthetic activity relative to the reduction in lipid peroxidation suggests that acclimation mechanisms are essential for minimizing oxidative stress damage and repairing the photosynthetic system after prolonged exposure to Cd. Elevated levels of POD can potentially enhance the elimination of H_2_O_2_, thereby promoting photosynthesis (Solti et al., 2016). Similarly, Alsherif et al. (2022) documented that *Amaranthus retroflexus* plants avoid ROS stress by increasing their antioxidant system, inducing lower HM harmful symptoms even at higher accumulation of HM levels.

In this study, PPO, POD, and CAT activities were observed to increase with moderate levels of Cd and Pb exposure, either individually or in combination (see **Figures 2A-C**). Wang et al. (2008) provided support for the increase in PPO, POD, and CAT activities relative to Cd and Pb treatments compared to the control. They noted that enzyme activities in metal accumulator plants escalated rapidly with rising levels of Cd. They also observed a swift increase in enzyme activities in metal accumulator plants as Cd levels increased. Increasing exposure time to HM stress in *P. nigra* L. led to a reduction in ROS, with increment of CAT and POD activities (Jakovljevic et al., 2014). Several studies provide insights into the dynamic responses of enzyme activity to HM exposure. Nagajyoti et al. (2010) observed that enzyme activity under HMs, such as Cu, Cd, and Pb, is concentration-dependent and can be either enhanced or reduced. Dai et al. (2012) investigated the response of *Populus canescens* and noted a significant increase in CAT activity at lower Cd concentrations (10 μM), while higher Cd levels (50 and 70 μM) led to a decrease in CAT activity across various tissues. Additionally, they observed that CAT activity followed a significant order: leaf > wood > bark > root. This suggests that *P. canescens* has well-established detoxification mechanisms to tackle metal-induced oxidative stress. Tauqeer et al. (2016) found that the POD and CAT activities in *A. bettzickiana* were enhanced at lower Cd and Pb (0.5 and 1.0 mM), and then their activities were retarded at higher Cd and Pb (2.0 mM). Zou et al. (2017) reported stimulation of CAT, POD, and SOD activities in *Salix matsudana* at Cd concentrations (10, 50, and 100 mM) but noted reductions in soluble protein and carbohydrate levels. Emamverdian et al. (2018) also observed enhancing enzymatic activities in *Indocalamus latifolius* under Pb at 500–2,000 mg/kg soil. Similarly, Bankaji et al. (2019) noted an increase in CAT activity in *Atriplex halimus* with increasing soil Pb levels from 0 to 600 mM. Mohamed et al. (2019) pointed out that total antioxidant activities were increased by 65.5% in 40 mg/L Pb-treated seedlings of *Jatropha curcas*, being higher compared to the control. Xu et al. (2019) highlighted variations in enzyme activity, suggesting regulation of Cd and Pb stress resistance by an integrated antioxidative system, with suppression of CAT and POD activities observed with increasing Cd and Pb concentrations in willow genotypes. El-Mahrouk et al. (2020) found that PPO, POD, and CAT activities in *P. nigra* were stimulated with CdCl_2_ at 20 and 40 mg/kg soil and with Pb acetate at 250 and 450 mg/kg soil, then decreased under medium and higher levels of CdCl_2_ (60 and 80 mg/kg soil) and Pb acetate (650 and 850 mg/kg soil). Wang et al. (2022) revealed that Cd at 12 mg/kg soil stimulated POD in Chinese sweetgum and POD activity in Chinese fir seedlings was stimulated under 6–24 mg Cd/kg; however, 36 mg of Cd/kg declined POD activities in both species relative to control. Additionally, 6 mg Cd/kg soil did not significantly enhance POD activity in *S. superba*. They highlighted that 12 mg Cd/kg soil increased CAT activity in *S. superba* and Chinese sweetgum seedlings, while Cd at 12–36 mg/kg decreased CAT activity in Chinese fir. Furthermore, Saleem et al. (2024) found that exposure to 50 and 100 µM Cd significantly increased H_2_O_2_ production and enzymatic antioxidant activity, as well as the levels of phenolic compounds, flavonoids, and proline in *P. sativum*. These collective findings provide valuable insights into the intricate interactions and mechanisms involved in heavy metal accumulation in plants. They serve to reinforce and support our observations on this subject.

Heavy metals cause injuries to cell walls and damage to membrane permeability; consequently, EL increases. Also, EL was increased by soil Cd, and Pb levels increased. EL occurs in various plant species, tissues, and kinds of cells and can be forced with all stressed conditions as HMs (Demidchik et al., 2003). The main cause of EL is pertinent and responsible for K^+^ efflux from plants’ cells through cation that continues from the plasma membrane (Demidchik et al., 2014). They added that programmed cell death (PCD) is caused by ROS that results from stress-induced EL. Under more stress, ROS increases K^+^ efflux through guard cell outward-rectifying K^+^ (GORK) channels, and the cells of plants lose so much K^+^ amount, which increases the activity of proteases and endonucleases and supports PCD. EL increased in *Brassica juncea* as a result of Cd at 200 mg/kg, elevating upon exposure time (Ahmad et al., 2016).

Moreover, the increment of Cd and Pb from 0.5 to 1.0 mM raised EL in *A. bettzickiana* (Tauqeer et al., 2016). A similar result was found in two genotypes of *P. sativum* (AG-10 and AP-3) under Cd treatment (Sager et al., 2020). El-Mahrouk et al. (2020) on *P. nigra* noticed that increasing CdCl_2_ from 20 to 80 mg/kg soil and Pb acetate from 250 to 850 mg/kg soil elevated EL, and it increased by increasing HM levels in the soil. Thus, reduction in growth traits, TI, and enzymatic activities may be due to the increase in EL under HM stress. However, under heavy metal stress, an inverse relationship exists between vegetative traits and TI, with an increase in enzyme activities and EL observed simultaneously. Conversely, the decrease in vegetative traits and TI, coupled with the increase in EL, can be ascribed to the detrimental effects of heavy metals, while the rise in enzyme activities signifies a defensive response to heavy metal stress.

Indeed, there is a relationship between the contents of HMs in the plant organs and their soil contents. The HM accumulation and content in different organs of plants have been affected by plant species, metal ion levels and kinds in the soil, soil pH, bioavailability of HMs, and root exudations (Zarubova et al., 2015). Similarly, Samuilov et al. (2016) found that the concentration of Pb in *Populus tremula* × *P. alba* organs is related to its content in soil. Various organs of *Salix polaris* treated with Cd have different Cd levels (Krajcarova et al., 2016). Furthermore, in *P. nigra* ‘Italica’ treated with Cd (10, 25, and 50 mg/kg soil) and Pb (400, 800, and 1,200 mg/kg soil), its roots have Cd and Pb more than its leaves and stems (Redovnikovic et al., 2017). Additionally, El Mahrouk et al. (2019, 2020) on *Salix mucronata* and *P. nigra*, respectively, concluded that the roots of the two species contained Cd and Pb compared to their leaves and stems when treated with CdCl_2_ (40–80 mg/kg soil) and Pb acetate (250–850 mg/kg soil). A study by Durante-Yanez et al. (2022) on *C. sericea* revealed that Cd and Pb contents in the roots were higher than in the leaves or stems, and Cd and Pb levels among various organs were significant. Accordingly, Rahman et al. (2022) showed that the seasonal elements in the leaves or barks of some tree species reduced in the order of Zn > Pb > Cu > Cd in industrial and residential areas, and the tree species significantly indicated various quantities of HM uptake. In addition, Yousefi et al. (2023) pointed out that a higher content of Cd was in the leaves and stems of *P. oleracea* treated with 25, 50, 75, and 100 mg/L Cd. Significant differences in nickel (Ni), copper (Cu), Cd, and Pb concentrations were observed between contaminated and uncontaminated plant organs of the genotypes of *S. alba* (347, NS 37/6, and B-44) and *S. viminalis* (Urošević et al., 2024). They reported that the roots accumulated the largest amounts of Ni and Pb, while Cd was accumulated in the leaves more than other *Salix* genotypes’ organs. This is supported by Kaur et al. (2024), who reported that *S. alba* roots accumulated Cd and Pb more than the stems and leaves, in the order of Cd > Pb > Cd + Pb.

In general, the levels of BCF_s_, or BCF_r_, are relative to the levels of metal, as well as the other HMs present in the soil. The current study showed a decreasing trend in Cd and Pb BCF with the upheaval of their levels in the soil. This was documented by Redovnikovic et al. (2017). Also, the BCF_s_ of Cd and Pb, when they were applied individually, were rather higher compared to when they were used in combination, having the same levels, except for the BCF_s_ of Pb; this may be due to the fact that they are being absorbed at lower levels as a result of the antagonism between them. This is in contrast to Cd or Pb BCF_r_, with the exception of Cd BCF_r_. *Conocarpus erectus* Cd BCF_s_ was >1, which matched with the findings of Dominguez et al. (2008), who showed that the Cd BCF of the leaves of *P. alba* was >1 (nearly 2) and the Cd BCF_s_ values of *P. alba* cv. 6K_3_ and *P. alba* cv. 14 P11 were 2.5 and 4, respectively (Zacchini et al., 2009). Also, the Cd BCF_s_ of *P. alba* was >1 and the Cd BCF_r_ values of *P. alba* and *Morus alba* were <1 at 40, 80, and 160 mg Cd/kg soil (Rafati et al., 2011). Previously, Migeon et al. (2009) mentioned that Cd BCF values were 1.39, 2.26, and 1.98 for *P. deltoides* × *P. nigra*, *P. tremula* × *P. tremuloides*, and *P. trichocarpa* × *P. deltoides*, in succession. Additionally, the Cd BCF values of poplar (Jakovljevic et al., 2013) and willow and poplar (Algreen et al., 2014) were >1. This was supported by Durante-Yanez et al. (2022), who concluded that the Cd BCF for *C. sericea* was >1. Our results indicated that Cd BCF_r_, Pb BCF_s_, and Pb BCF_r_ values were <1. The findings are in agreement with those of Redovnikovic et al. (2017), who showed that the Cd and Pb BCF values for poplar under 10, 25, and 50 mg Cd/kg soil and 400, 800, and 1200 mg Pb/kg soil were <1, and the Pb BCF reached 0.23 with the highest Pb content. This was supported by Algreen et al. (2014) for the Pb BCF of willow and poplar trees at Danish polluted sites with HMs. Soil pH, EC, and Ca levels significantly affected the bioavailability of Pb (Zhang et al., 2019). Also, the Cd BCF_r_ and Pb BCF values of the leaves, stems, and roots of *S. mucronata* and *P. nigra* grown in soil containing CdCl_2_ (20–80 mg/kg) and Pb acetate (250–850 mg/kg) were <1 (El-Mahrouk et al., 2019, 2020).

The movement of metal ions is more related to the efficiency of plants for metal uptake, the movement of metal ions through the intracellular system, metal accumulation in various plant organs, and soil metal level. The Cd and Pb transfer within the plant can be affected by cellar displacement, which affects the free concentrations (Niu et al., 2007; Fuleker et al., 2009; Pourrut et al., 2011). Plant mechanisms such as adjusting membrane permeability, modifying cell wall capacity, or increasing the secretion of chelating compounds play crucial roles in maintaining optimal physiological levels of essential nutrients. These mechanisms help mitigate the toxic effects of HMs on aboveground organs when plants are exposed to such stresses (Kalaivanan and Ganeshamurthy, 2016). The least soil Cd and Pb levels may be a crucial factor for the roots’ actual accumulation quantities, which helps in the transference of metals through detoxification mechanisms and development in the plant obstructing ions in the vacuole through adhesion and union with ligands (proteins, organic acids, and peptides) leading to higher translocation amounts (Nworie and Lin, 2021; Sharma et al., 2021). Similarly, Rafati et al. (2011) treated *P. alba* and *M. alba* with 40–160 mg/kg soil, and they found that the Cd TF of the two species was >1. Also, Redovnikovic et al. (2017) found that *P. nigra* cv. ‘Italica’ grown in soil polluted with 25 and 50 mg Cd/kg and Pb at 800 and 1,200 mg/kg recorded Cd TF >1 and Pb <1. In addition, the Pb TF was <1 for *P. nigra* and *C. sericea* grown under various concentrations of Pb (El-Mahrouk et al., 2020; Durante-Yunez et al., 2022). Additionally, the BCF and TF of Cd were higher than those of Pb in all *S. alba* organs, and the combined utilization of Cd and Pb affected BCF at each application level, demonstrating a negative interaction between Cd and Pb (Kaur et al., 2024). Based on the data related to TI, Cd contents in plant organs, BCF_s_, BCF_r_, and TF, *C. erectus* is a suitable species for Cd phytoextraction under all the Cd treatments used because its BCF_s_ and TF were >1, and it is a phytoextractor for Pb at (L) Pb, (M) Pb, and (M) Cd (M) Pb treatments because its BCF_r_ was <1 and its TF was >1. Also, *C. erectus* is a phytostabilizator for Pb at (H) Рb, (L) Cd (L) Pb, and (H) Cd (H) Pb treatments because its Pb BCF_r_ and TF were <1. These results were supported by the correlation of data among Cd or Pb parameters.

## 5 Conclusion

The issue of heavy metal contamination in agricultural soils is a significant challenge encountered by numerous countries globally. Resolving this issue necessitates the utilization of safe and eco-friendly methods, such as phytoremediation, that do not exert adverse effects on the environment. The identification of novel plant species, like *C. erectus*, is crucial, as it has exhibited notable tolerance to Cd and Pb at levels reaching 60 mg/kg and 700 mg/kg in soil, respectively. Generally, HMs have a detrimental impact on plant development, biochemical structure, and EL due to their accumulation in plant tissues. *Conocarpus erectus* is considered a strong Cd accumulator because its Cd BCF_s_ and Cd TF were >1 in all tested Cd levels. Additionally, *C. erectus* is suitable for Pb phytoextraction at 400 and 700 mg Pb/kg soil and 60 mg Cd + 700 mg Pb/kg soil, as its BCF of the shoots or roots were <1 and its TF were >1. Furthermore, *C. erectus* is appropriate for Pb phytostabilization at 1,000 mg Pb, 40 mg Cd + 400 mg Pb, and 80 mg Cd + 1000 mg Pb/kg soil, where the Pb BCF of the shoots or roots and the Pb TF were <1. There is a highly significant positive correlation between vegetative traits and the tolerance index of *C. erectus*. Conversely, under Cd and Pb levels, the correlation between vegetative traits (which decrease) and enzyme activities or EL (which increased) is highly significantly negative. Moving forward, the forthcoming study should incorporate interventions such as microbe-assisted techniques, organic amendments, biochar application, and natural extracts to enhance the tolerance mechanisms of plant species and to understand the influences of HMs on the foliage and xylem structure as well as the chemical compositions such as tannins and phenols found in the wood of *C. erectus.* Moreover, the research should ascertain the quantities of cellulose, hemicellulose, lignin, and ash present under the influence of HM stress.

## Data Availability

The original contributions presented in the study are included in the article/supplementary material. Further inquiries can be directed to the corresponding authors.
